# Daily Supplementation of L-Glutamine in Atrial Fibrillation Patients: The Effect on Heat Shock Proteins and Metabolites

**DOI:** 10.3390/cells9071729

**Published:** 2020-07-20

**Authors:** Roeliene Starreveld, Kennedy S. Ramos, Agnes J. Q. M. Muskens, Bianca J. J. M. Brundel, Natasja M. S. de Groot

**Affiliations:** 1 Department of Cardiology, Erasmus University Medical Center, 3015 GD Rotterdam, The Netherlands; r.starreveld@erasmusmc.nl (R.S.); k.silvaramos@amsterdamumc.nl (K.S.R.); a.muskens@erasmusmc.nl (A.J.Q.M.M.); 2 Department of Physiology, Amsterdam UMC, Vrije Universiteit Amsterdam, Amsterdam Cardiovascular Sciences, 1081 HV Amsterdam, The Netherlands; b.brundel@amsterdamumc.nl

**Keywords:** atrial fibrillation, glutamine, heat shock proteins, metabolites, therapy

## Abstract

Pharmaco-therapeutic strategies of atrial fibrillation (AF) are moderately effective and do not prevent AF onset and progression. Therefore, there is an urgent need to develop novel therapies. Previous studies revealed heat shock protein (HSP)-inducing compounds to mitigate AF onset and progression. Such an HSP inducing compound is L-glutamine. In the current study we investigate the effect of L-glutamine supplementation on serum HSP27 and HSP70 levels and metabolite levels in patients with AF patients (*n* = 21). Hereto, HSP27 and HSP70 levels were determined by ELISAs and metabolites with LC-mass spectrometry. HSP27 levels significantly decreased after 3-months of L-glutamine supplementation [540.39 (250.97–1315.63) to 380.69 (185.68–915.03), *p* = 0.004] and normalized to baseline levels after 6-months of L-glutamine supplementation [634.96 (139.57–3103.61), *p* < 0.001]. For HSP70, levels decreased after 3-months of L-glutamine supplementation [548.86 (31.50–1564.51) to 353.65 (110.58–752.50), *p* = 0.045] and remained low after 6-months of L-glutamine supplementation [309.30 (118.29–1744.19), *p* = 0.517]. Patients with high HSP27 levels at baseline showed normalization of several metabolites related to the carbohydrates, nucleotides, amino acids, vitamins and cofactors metabolic pathways after 3-months L-glutamine supplementation. In conclusion, L-glutamine supplementation reduces the serum levels of HSP27 and HSP70 within 3-months and normalizes metabolite levels. This knowledge may fuel future clinical studies on L-glutamine to improve cardioprotective effects that may attenuate AF episodes.

## 1. Introduction

Atrial fibrillation (AF) is a progressive, age-related disease affecting worldwide approximately 33.5 million individuals and is therefore regarded as one of the cardiovascular epidemics of the 21th century [1]. AF is associated with severe complications such as thrombo-embolic events, impaired cognitive function and even increased mortality [2].

At present, treatment modalities for AF are only moderately effective and do not prevent AF onset and progression from recurrent intermittent episodes to finally permanent AF. Though invasive ablation therapy initially seemed promising in early stage AF, up to 50% of the AF patients with more persistent types of AF have recurrences within 1 year and require multiple procedures [3]. Pharmacological therapy of AF is even less effective, and its usage is limited by severe and potentially life-threatening side-effects. Main reason for AF therapy failure is that pharmaco-therapeutic strategies are not directed at mechanistic root causes of AF, which drive structural cardiomyocyte damage, mitochondrial dysfunction and consequently electrical and contractile dysfunction of atrial cardiomyocytes [4,5].

Emerging research findings indicate that heat shock proteins (HSP) mitigate AF onset and progression in experimental model systems for AF [6,7,8,9]. Especially HSP27 (small HSP) has shown promising protective effects against AF in-vitro [10]. Importantly, patients with persistent AF reveal exhaustion of HSP27 and HSP70, and not HSP40 and HSP90, atrial tissue levels when compared to control patients in sinus rhythm. In line, pharmacological induction of HSP levels with HSP-inducing compounds attenuated AF promotion in dog models for atrial tachypacing and ischemia-induced AF [10,11]. Evidence reveals that L-glutamine, a semi-essential amino acid, represents a potent inducer of intracellular HSP levels, which has been progressively investigated in cardiovascular research. Clinical and experimental studies have consistently presented increased intracellular expression of HSPs, especially HSP27 and HSP70, after L-glutamine administration [12,13,14]. Given the fact that human body levels of L-glutamine are rapidly metabolized and in deficit under catabolic conditions [15], L-glutamine supplementation resulted in attenuation of experimental cellular injury and in prognostic amelioration of critically ill patients diagnosed with sepsis, heart failure and patients undergoing cardiopulmonary bypass surgery [14,16,17,18,19]. In prior studies, it was shown that L-glutamine enhances translocation of trimerized phosphorylated HSF1 from cytosol to the nucleus, followed by increased DNA binding to heat shock elements in the promotor sequence of *hsp* genes, thereby resulting in increased HSP expression [20]. Increased HSP expression promotes enhancement of contractile function and prevents loss of cellular integrity during AF [9]. In addition to a role in HSP expression, L-glutamine and its metabolites are important in ATP, DNA and nucleotide formation, suppression of inflammation, attenuation of oxidative stress and apoptosis, and increased blood fluidity and flow [21,22,23,24,25]. Although the role of L-glutamine as an inducer of HSP expression and protector against various diseases including ischemic heart disease and heart failure has been recognized [17,18], its potential protective role in AF progression has not been investigated.

Based on experimental and clinical pilot studies, we hypothesize that L-glutamine suppletion alters blood-based HSP levels and improves the metabolic stage of AF patients. In this pilot study, we therefore measured the effect of L-glutamine on serum HSP levels, and studied the influence on serum metabolite levels in AF patients. This knowledge may fuel future clinical studies on L-glutamine to attenuate AF episodes.

## 2. Materials and Methods

This prospective interventional trial was part of the Glutamine Suppletion Minimizes the Atrial Fibrillation Burden (Glutaminimize AF) project, which was approved by the local ethics committee in the Erasmus University Medical Center Rotterdam (MEC-2017-524). Written informed consent was obtained from all patients.

### 2.1. Study Population

Patients with diagnosed AF and frequent symptoms of AF episodes (≥ 1/week) were recruited at the outpatient clinic of the Cardiology department. Patients with diabetes mellitus or a soya, gluten or shellfish allergy were excluded. Patients were instructed to maintain their regular diet during the whole study. Patient characteristics (e.g., age, medical history, cardiovascular risk factors, type of AF) were obtained from the patient’s medical records.

### 2.2. Study Design

**Figure 1:**
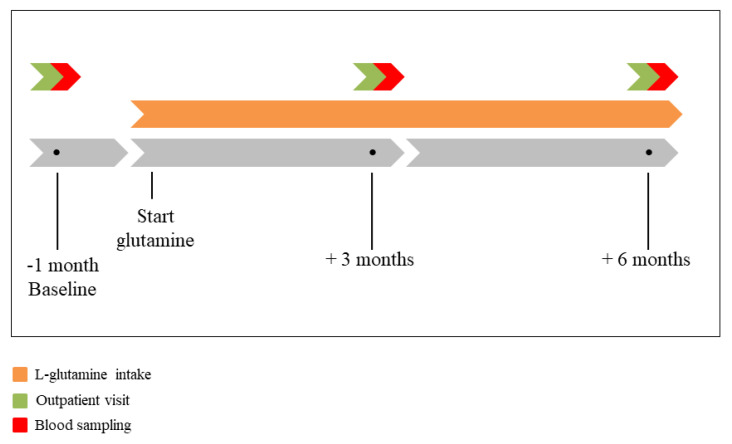
Time course of the Glutaminimize AF project. At baseline, 3-month and 6-month follow-up all patients visited the outpatient clinic (green bar) for electrocardiography and blood sampling (red bar). After one month, patients started with oral intake of the KABI^®^ Glutamine sachets containing 10 g of L-glutamine (orange bar), twice daily for a period of six months.

Patients started with oral intake of the KABI^®^ Glutamine sachets containing 10 g of L-glutamine, twice daily (once in the morning, once in the evening) for a period of six months. Patients were evaluated at a dedicated outpatient clinic at baseline and at three and six months after start of L-glutamine intake (Figure 1). During those visits, blood was drawn for testing of HSP levels and metabolomics.

### 2.3. Data Analysis

#### 2.3.1. HSP Measurements

Immediately after blood sample collection, serum was harvested from blood in BD Vacutainer™ SST™ II Advance Tubes (Fisher Scientific) by centrifugation at 2000× *g* for 10 min at 4 °C and frozen in −80 °C until analysis of HSP27 and HSP70. For measurement of serum HSP27 levels, samples were diluted six times and for HSP70 levels samples were diluted twice in 1% BSA in PBS. The amount of HSP27 and HSP70 protein was detected in triplicates using human HSP27 or HSP70 DuoSet^®^ ELISA kits from R&D Systems (Cat. no. DY1580 and DY1663, respectively) according to the manufacturer’s instructions with minor adjustments (serum was incubated at 4 °C overnight, instead of 2 h at room temperature). The HSP levels, as well as the difference in HSP27 or HSP70 levels between follow-up moments, were the primary study parameters.

#### 2.3.2. Metabolomics

For a subanalysis, mass spectrometry was used to derive metabolite levels from four pooled patients with the highest HSP27 levels and five pooled patients with the lowest HSP27 levels at baseline. The metabolite levels of the same pooled patients at 3-month follow-up were also obtained. Metabolite measurements from the four mentioned pooled samples were used to determine the effect of L-glutamine supplementation on various metabolites levels. Ratios were derived between the pooled metabolites of high HSP27 at baseline and 3-months of L-glutamine supplementation (high HSP: 3M/baseline), low HSP27 at baseline and 3-months of L-glutamine supplementation (low HSP: 3M/baseline) and high HSP27 and low HSP27 at baseline (baseline: low HSP/high HSP).

Determination of metabolites was performed as previously described, with minor adjustments [26]. A 75 µL mixture of internal standards in water, as listed in the Appendix A, was added to each sample. Subsequently, 425 µL water, 500 µL methanol and 1 mL chloroform were added to the same 2 mL tube before thorough mixing and centrifugation for 10 min at 14,000 rpm. The top layer, containing the polar phase, was transferred to a new 1.5 mL tube and dried using a vacuum concentrator at 60 °C. Dried samples were reconstituted in 100 µL methanol/water (6/4; *v/v*). Metabolites were analyzed using a Waters Acquity ultra-high-performance liquid chromatography system coupled to a Bruker Impact II™ Ultra-High Resolution Qq-Time-Of-Flight mass spectrometer. Samples were kept at 12 °C during analysis and 5 µL of each sample was injected. Chromatographic separation was achieved using a Merck Millipore SeQuant ZIC-cHILIC column (PEEK 100 × 2.1 mm, 3 µm particle size). Column temperature was held at 30 °C. Mobile phase consisted of (A) 1:9 acetonitrile:water and (B) 9:1 acetonitrile:water, both containing 5 mM ammonium acetate. Using a flow rate of 0.25 mL/min, the LC gradient consisted of: 100% B for 0–2 min, ramp to 0% B at 28 min, 0% B for 28–30 min, ramp to 100% B at 31 min, 100% B for 31–35 min. MS data were acquired using negative and positive ionization in full scan mode over the range of *m/z* 50–1200. Data were analyzed using Bruker TASQ software version 2.1.22.3. All reported metabolite intensities were normalized to internal standards with comparable retention times and response in the MS. Metabolite identification has been based on a combination of accurate mass, (relative) retention times and fragmentation spectra, compared to the analysis of a library of standards.

### 2.4. Statistical Analysis

Data was tested for normality. Normally distributed continuous variables were expressed as mean ± SD, skewed continuous variables were expressed as median (minimum-maximum) and categorical variables were expressed as numbers (percentages). Depending on skewness of the data, a paired-samples T test or Wilcoxon signed-rank test was used to compare HSP levels between each of the three follow-up moments (i.e., baseline, 3-month and 6-month follow-up). Bonferroni correction was applied for comparison of the three measurements; a *p*-value of < 0.0167 (0.05/3) was considered statistically significant. Correlations between clinical variables were calculated using Pearson’s or Spearman’s correlation coefficient, depending on skewness of the data. In general, a *p*-value < 0.05 was considered statistically significant. Metabolite ratios higher than 1.5 were classified as increased, whereas ratios lower than 0.667 were classified decreased. Metabolite ratios between 0.667 and 1.5 were classified as stable. All statistical analyses were performed using R Statistical Software (RStudio, Inc., Boston, MA, USA; version 1.0.153).

## 3. Results

### 3.1. Study Population

Clinical characteristics of all patients (*n* = 21, 16 male (76.2%), age 58.7 ± 10.5 years) are presented in Table 1. Patients had a history of paroxysmal (*n* = 13, 61.9%), persistent (*n* = 6, 28.6%) or longstanding persistent AF (*n* = 2, 9.5%). The far majority of patients had a normal left ventricular function (*n* = 18, 85.7%). All patients completed the 3-month follow-up (*n* = 21, 100%), whereas 20 patients (95.2%) completed the 6-month follow-up.

### 3.2. Effect of L-Glutamine on HSP Levels

**Figure 2:**
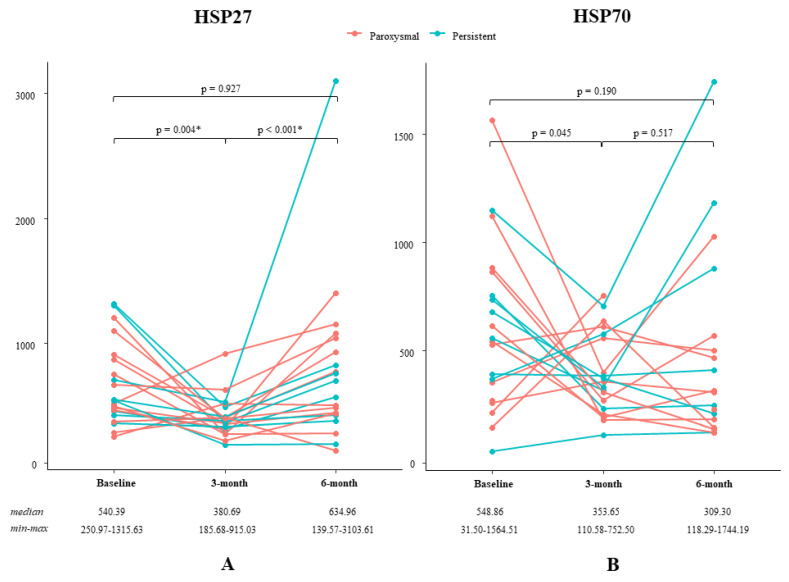
Effect of glutamine on HSP levels. (**A)**: Levels of HSP27 per patient during the study period. (**B)**: Levels of HSP70 per patient during the study period. Statistical significance is indicated with an asterisk (*, *p* < 0.0167).

Median HSP27 level at baseline was 540.39 and ranged from 250.97 to 1315.63. As seen in Table 2 and Figure 2, levels of HSP27 significantly decreased to 380.69 (185.68–915.03) after 3-months of L-glutamine supplementation (*p* = 0.004). After 6-months of L-glutamine supplementation, the level of HSP27 normalized to baseline levels [634.96 (139.57–3103.61), *p* < 0.001]. For HSP70, the level at baseline was 548.86 (31.50–1564.51) and decreased to 353.65 (110.58–752.50) after 3-months of L-glutamine supplementation (*p* = 0.045). The HSP70 level remained low after 6-months of L-glutamine supplementation [309.30 (118.29–1744.19), *p* = 0.517]. There were no significant differences between the HSP27 and HSP70 levels at baseline and after 6-months of L-glutamine use. Unfortunately, HSP70 levels of patient 8 (baseline, 3-month and 6-month follow-up), patient 14 (6-month follow-up) and patient 15 (3-month follow-up) could not be determined due to poor quality of the sample.

**Figure 3:**
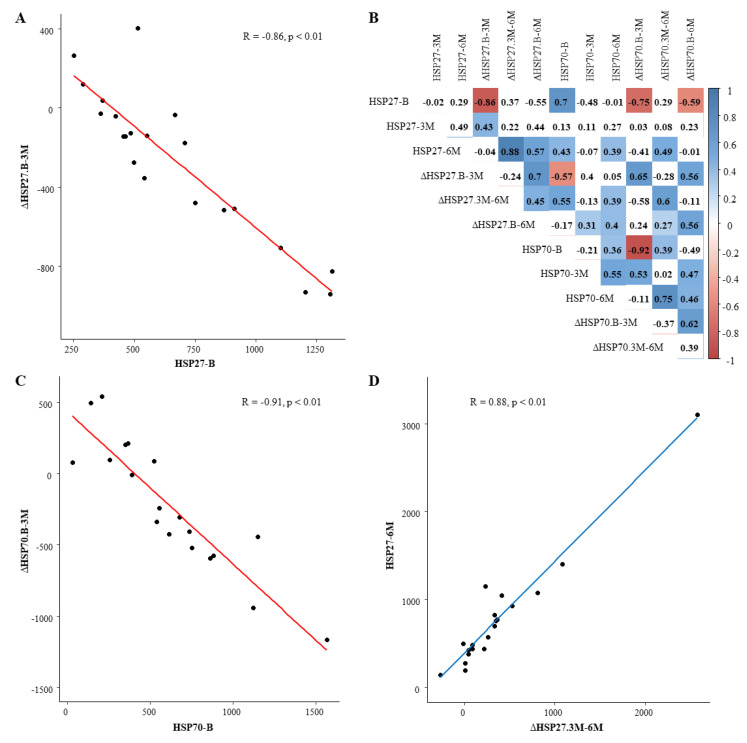
Relation between (∆) HSP27 and/or (∆)HSP70 levels during the study period. (**A**): Scatterplot with regression line for the relation between HSP27 at baseline and the ∆HSP27 from baseline to 3-month follow-up. The corresponding correlation coefficient and statistical significance are indicated in the graph. (**B**): Correlogram with correlation coefficients for all possible relations between (∆) HSP27 and/or (∆) HSP70. Significant correlations (*p* < 0.05) are colored with either blue (positive correlation) or red (negative correlation). Variables with a strong correlation (−0.8 < R > 0.8) are visualized in the other panels. (**C**): Scatterplot with regression line for the relation between HSP70 at baseline and the ∆HSP70 from baseline to 3-month follow-up. (**D**): Scatterplot with regression line for the relation between ∆HSP27 from 3-month to 6-month follow-up and HSP27 at 6-month follow-up.

The relation between (∆)HSP27 and/or (∆)HSP70 levels at baseline and after 3-months and 6-months of L-glutamine supplementation is illustrated in Figure 3. The level of HSP27 at baseline showed a strong negative correlation with the ∆HSP27 from baseline to 3-months L-glutamine supplementation (R = - 0.86, *p* < 0.001, upper left panel). Similarly, a strong negative correlation was found between the level of HSP70 at baseline and the ∆HSP70 from baseline to 3-months L-glutamine supplementation (R = - 0.91, *p* < 0.001, lower left panel). In addition, a strong positive correlation between the ∆HSP27 from 3-months to 6-months L-glutamine supplementation and the HSP27 at 6-month L-glutamine supplementation was found (R = 0.88, *p* < 0.001, lower right panel). At baseline, levels of HSP27 and HSP70 showed a significant positive correlation (R = 0.7, *p* < 0.001, upper right panel).

### 3.3. Relation between HSP Levels and Patient Characteristics

**Figure 4:**
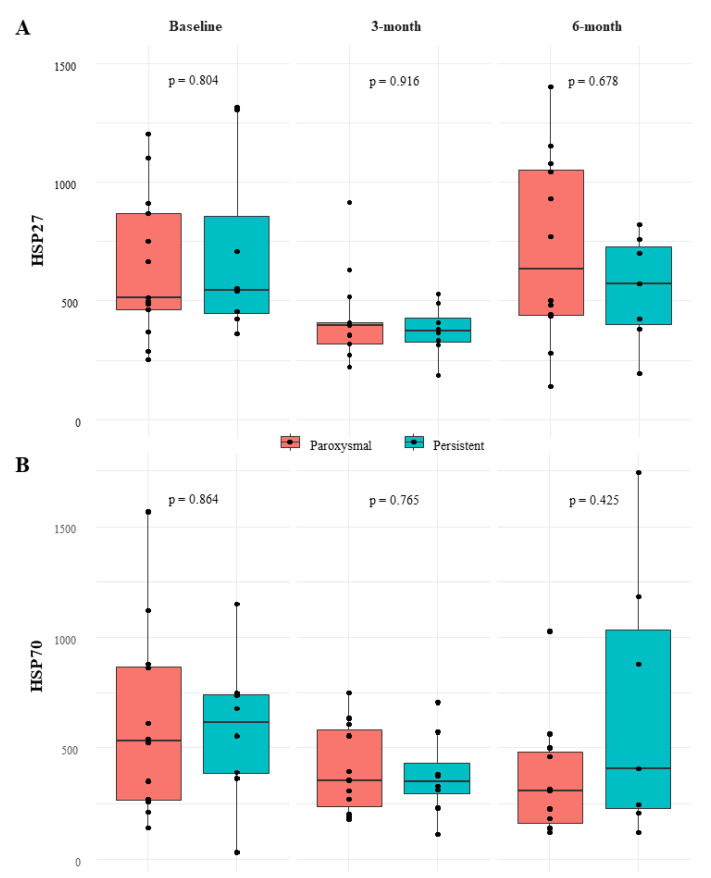
HSP levels for paroxysmal and (longstanding) persistent AF patients. (**A**): Boxplots of HSP27 levels for paroxysmal and (longstanding) persistent AF patients separately during the study period. (**B**): Boxplots of HSP70 levels for paroxysmal and (longstanding) persistent AF patients separately during the study period.

Levels of HSP27 and HSP70 did not differ between patients with paroxysmal or (longstanding) persistent AF, as illustrated in Figure 4. In addition, no statistical difference was found in HSP27 and HSP70 levels between male and female patients, and HSP27 and HSP70 levels did not correlate with L-glutamine compliance nor with age.

### 3.4. Relation between HSP27 and Energetic Metabolism

A positive correlation between HSP27 and HSP70 levels at baseline was observed. Given the highly significant reduction of HSP27 during 3-months of L-glutamine supplementation, further metabolite analysis was performed based on HSP27 levels. Hereto, the effect of 3-months L-glutamine supplementation on metabolite levels from patients showing high HSP27 levels was contrasted to low HSP27 levels at baseline. Mass spectrometry was utilized i pooled serum samples, as described in the Methods.

**Figure 5:**
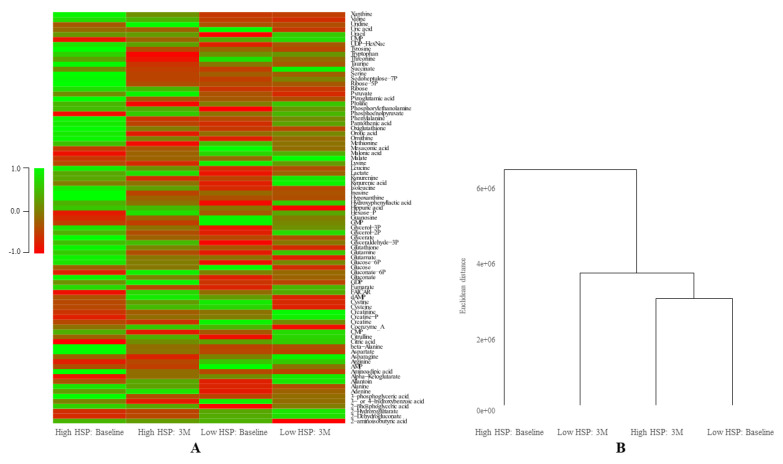
HSP level and L-glutamine supplementation affects metabolic phenotypes. (**A**): Heat map of the absolute levels of all 83 metabolites for high HSP: baseline, high HSP: 3-month follow-up (3M), low HSP: baseline and low HSP: 3M. The pooled group high HSP at baseline is distinct from the other groups. (**B**): Dendrogram showing the hierarchical clustering using the Euclidean distance with average linkage for all four groups.

In total 83 metabolites were identified in the four groups. The derived ratios between the groups are listed in Table S1. A heat map and dendrogram generated from the absolute metabolite levels showed that the group with high HSP at baseline is distinct from the other three groups, suggesting that high HSP levels at baseline represent a different metabolic phenotype compared to low HSP levels at baseline and after 3-months of L-glutamine supplementation (Figure 5).

Based on the ratios (i.e., high HSP: 3M/baseline, low HSP: 3M/baseline, baseline: low HSP/high HSP), metabolites were clustered, as listed in Table 3. In total, 55.4% of metabolite levels were comparable between the four groups. In high HSP levels at baseline, seven metabolites (8.43%) showed normalization after 3- months L-glutamine supplementation and were comparable to low HSP levels at baseline and 3- months of L-glutamine supplementation. For both high and low HSP at baseline, two metabolites (2.4%) showed a simultaneous increase (ratios >1.5) and one metabolite (1.2%) showed simultaneous decrease (ratios <0.667) after 3- months L-glutamine supplementation. The remaining metabolites, 31.3%, did show level changes in ratios (<0.667 or >1.5), which could partly explain the hierarchical clustering as observed in Figure 5.

**Figure 6:**
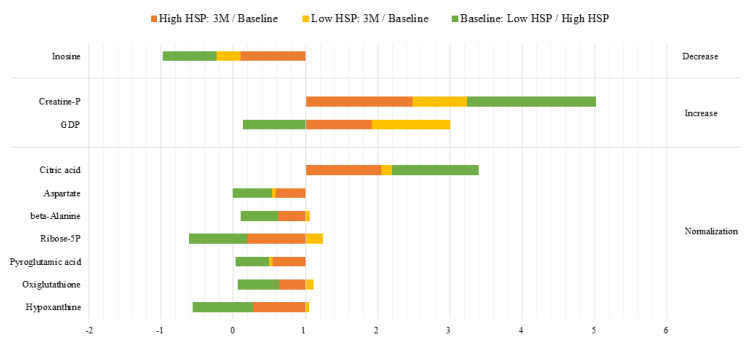
Metabolite ratios of high HSP: 3M/baseline, low HSP: 3M/baseline and baseline: low HSP/high HSP, grouped on behavior. Inosine showed simultaneous decrease (ratio < 0.667), whereas Creatine-P and GDP showed simultaneous increase (ratio > 1.5) in both high HSP: 3M/baseline and low HSP: 3M/baseline, representing the effect of L-glutamine supplementation on both. Seven metabolites (8.43%) showed a normalization behavior, in which high HSP: 3M/baseline shows a normalization (either increase or decrease) towards stable levels of low HSP: 3M/baseline.

By contrasting the metabolic changes between the groups, key metabolites were identified: ribose-5P, aspartate, beta-alanine, hypoxanthine, oxiglutathione, pyroglutamic acid, citric acid, creatine P, GDP and inosine (Figure 6). In Table S2, all human pathways and global pathways related to these metabolites are listed. Findings indicate that 3-months L-glutamine supplementation affect pathways related to carbohydrates, nucleotide, amino acid and vitamin synthesis.

## 4. Discussion

Despite many innovative insights and interventions in recent years, still no curative therapy for AF patients exists. Our pilot study is the first to investigate the effect of L-glutamine on HSP27 and HSP70 levels in serum samples of AF patients. After 3-months of L-glutamine supplementation, the level of HSP27 and HSP70 significantly decreased, and HSP27 normalized to baseline levels after 6-months of L-glutamine supplementation. A strong correlation was found between the baseline level of HSP27 or HSP70 and the degree of reduction at 3-months of L-glutamine supplementation: patients with a higher baseline level of HSP27 or HSP70 revealed a large reduction, whereas patients with a low HSP27 or HSP70 level at baseline stayed low after 3-months supplementation. Analysis of metabolites revealed that 3-months of L-glutamine supplementation normalized the levels of metabolites related to the synthesis of carbohydrates, nucleotides and amino acids, compared to non-treated patients with high levels of HSP at baseline. These findings indicate that 3-months L-glutamine supplementation reduces HSP27 and HSP70 levels in serum samples which is accompanied with normalization of metabolites of fundamental pathways within cell function.

### 4.1. Effect of L-glutamine on HSP and Metabolite Levels

Recent experimental studies by Brundel et al. showed that AF is associated with structural damage in the cardiomyocyte due to derailment in proteostasis (i.e., protein expression, function and clearance), a process which could be normalized by overexpression of HSP27 [6,7,10,27]. Various HSPs, including the HSP27 and HSP70, are involved in the protection against different forms of cellular stress by functioning as intra-cellular chaperones for other proteins [6,28]. In cardiomyocytes, HSP27 can bind to structural proteins and thereby shield them from damage and functional loss [6,9,10]. In accordance, upregulation of HSP27 in atrial tissue of dogs protected cardiomyocytes from AF-induced cellular stress and as such contributed to the maintenance of atrial tissue integrity and contractile function [6,28]. These findings indicate that pharmacological induction of HSPs may be an interesting target to treat AF. Previous studies by Gong and Jing, Hamiel et al. and Hayashi et al. demonstrated L-glutamine to induce expression of HSPs in organs, including the heart [1,2,3,4,5,6,7,8,9,10,11,12,13,14]. The precise interaction between induced levels of HSPs in cardiac tissue and its effect on HSP levels in serum is not fully known. Our data demonstrate HSP27 and HSP70 to decrease from baseline to 3-months of L-glutamine supplementation, followed by a partly increase from 3-months to 6-months of L-glutamine supplementation. Although several studies have detected HSP27 and HSP70 in the extracellular milieu, there is still lack of consensus regarding the exact mechanisms of transmembrane transport, as well as their role outside the cell of origin [29]. Given that HSP’s function of protein homeostasis belongs to the intracellular space, L-glutamine administration may firstly increases intracellular levels of HSPs, thereby promoting enhancement of contractile function during AF. This beneficial state may prevent cellular disruption and subsequent pathological release of HSP from the cardiomyocytes into the circulation as observed in the period from baseline to 3-months. Further studies are necessary to reveal a mechanistic understanding of the interaction between cardiomyocyte and serum levels of HSPs after L-glutamine supplementation and the duration of the potential beneficial effect on HSP levels. Unfortunately, in the current study no atrial tissue samples could be selected, and therefore the effect of L-glutamine on atrial tissue and serum HSP levels could not be studied. Although previous studies indicate an increase in HSPs in serum and tissue after L-glutamine administration [30,31,32,33], very few studies measured HSPs in human cardiac tissue, specifically atrial tissue, nor performed long-term usage of L-glutamine.

Interestingly, patients with a higher HSP27 or HSP70 baseline level decreased more in HSP level during 3-months of L-glutamine supplementation in comparison to patients with a low HSP27 or HSP70 baseline level, which stayed low at 3-months L-glutamine supplementation. In correspondence, our findings revealed normalization of several metabolites to levels as observed in patients with low HSP at baseline and over the course of 3-months L-glutamine supplementation. This suggests a beneficial effect of L-glutamine on these metabolites, which may reveal an influence on carbohydrates, nucleotides, amino acids and cofactors and vitamins metabolism. In addition, a simultaneous and parallel variation—increase or decrease—in high HSP and low HSP throughout the 3-months of L-glutamine supplementation was observed for metabolites related to amino acids and nucleotide metabolism. These findings indicate that L-glutamine supplementation reveals an influence on metabolic phenotypes, encouraging further research on its influence on energy metabolic status in AF patients.

### 4.2. Study Limitations and Future Perspectives

The small sample size of this prospective interventional pilot trial disables the formulation of strong conclusions. Also, patients with paroxysmal as well as (longstanding) persistent AF of varying age and co-morbidities participated in this study, potentially leading to (unaccounted) differences in individual responses to administration of L-glutamine. Nevertheless, a (significant) reduction in HSP27 and HSP70 serum levels was observed after L-glutamine supplementation, substantiating a possible effect of L-glutamine on HSP serum levels. Further mechanistic investigation is warranted to elucidate the exact relation between HSPs levels in atrial tissue and in blood. Therefore, it is of interest to mount a future clinical study to test a beneficial effect of L-glutamine on the reduction of AF burden. Hereto, we recommend a large – ideally randomized controlled – trial using continuous rhythm monitoring to determine the AF burden together with a validated patient-observed-effect questionnaire, in order to gain insight into the (patient-specific) effects of L-glutamine on HSP levels and AF burden. In such a trial, individual metabolomics analyses could be performed to reveal the metabolic phenotype of each patient, allowing accurate correlation with clinical parameters for AF progression.

## 5. Conclusions

Our study reveals that the level of HSP27 significantly decreased after 3-months of L-glutamine supplementation, and partly normalized to baseline levels after 6-months of L-glutamine supplementation. A strong correlation was found between the baseline level of HSP27 or HSP70 and the amount of decrease at 3-months of L-glutamine supplementation: patients with a higher baseline level of HSP27 or HSP70 decreased more, whereas patients with a low HSP27 or HSP70 level remained low after 3-months supplementation. Analysis of metabolites revealed that several metabolites related to carbohydrates, nucleotides, amino acids vitamins metabolism pathway are normalized in patients with high level of HSP27 at baseline, indicating a potential beneficial effect of L-glutamine on the metabolic profile. Next step is to mount a clinical trial in AF patients and study the effect of L-glutamine on AF burden.

## Figures and Tables

**Table 1:** Patient characteristics.

Number of patients	21
Male	16 (76.2)
Age (years)	58.7 ± 10.5
BMI	26.7 (22.0–36.3)
Type of AF:	
Paroxysmal	13 (61.9)
Persistent	6 (28.6)
Longstanding persistent	2 (9.5)
Time since AF diagnosis (years)	1.8 (0.2–19.5)
Hypertension	6 (28.6)
Dyslipidemia	2 (9.5)
Left ventricular function:	
Normal	18 (85.7)
Mild impairment	3 (14.3)
Use of anti-arrhythmic drugs (at baseline):	
Class I	8 (38.1)
Class II	6 (28.6)
Class III	7 (33.3)
Class IV	3 (14.3)
Class V	1 (4.8)
Follow-up duration:	
Completed 3-month follow-up	21 (100)
Completed 6-month follow-up	20 (95.2)
Glutamine compliance:	
Baseline to 3-month follow-up (*n* = 20)	0.99 (0.77–1.00)
3-month to 6-month follow-up (*n* = 19)	0.97 (0.61–1.00)
Baseline to 6-month follow-up (*n* = 20)	0.97 (0.69–1.00)
Reduced kidney function (*n* = 17)	1 (5.9)
Reduced liver function (*n* = 17)	0 (0)
Reduced thyroid function (*n* = 17)	0 (0)

Values are presented as N (%), mean ± SD or median (min-max), whichever appropriate. Glutamine compliance is calculated as the ratio of actual glutamine intake and the prescribed regimen. BMI = body mass index.

**Table 2:** Serum levels of HSP27 and HSP70 at baseline (B), 3-month (3M) and 6-month (6M) follow-up. L-PER = longstanding persistent AF, NA = not available, PAR = paroxysmal AF, PER = persistent AF.

Study ID	AF Type	HSP27-B	HSP27-3M	HSP27-6M	HSP70-B	HSP70-3M	HSP70-6M
1	L-PER	1315.63	489.79	821.92	751.86	230.31	245.39
2	L-PER	455.71	312.78	571.25	31.50	110.58	118.29
3	PAR	869.73	352.14	441.98	862.09	268.50	565.83
4	PAR	751.50	271.66	278.59	542.14	202.70	120.66
5	PAR	288.50	407.61	769.92	524.28	610.66	463.72
6	PAR	514.07	915.03	1150.48	258.42	353.65	304.15
7	PAR	250.97	516.21	501.01	352.67	555.83	500.92
8	PAR	463.89	319.85	1401.11	NA	NA	NA
9	PAR	1203.41	271.80	1078.15	1564.51	396.58	1027.39
10	PAR	485.48	356.38	-	211.71	752.50	-
11	PER	360.65	332.83	379.69	390.49	382.47	409.58
12	PER	706.81	528.21	3103.61	1149.16	705.89	1744.19
13	PER	423.98	380.69	421.96	365.78	574.18	878.51
14	PER	1305.84	364.30	698.66	555.57	312.52	NA
15	PAR	666.37	629.06	1044.07	1119.96	177.28	179.68
16	PAR	911.61	402.25	929.48	614.61	190.27	314.44
17	PAR	369.99	407.58	139.57	143.11	635.17	142.24
18	PER	550.98	409.01	758.89	735.83	328.16	1182.55
19	PAR	1102.87	394.93	483.66	880.77	304.32	135.65
20	PER	540.39	185.68	192.68	678.67	371.17	208.70
21	PAR	498.64	221.64	437.17	268.38	NA	225.46
Median	-	540.39	380.69	634.96	548.86	353.65	309.30
Min-Max	-	250.97–1315.63	185.68–915.03	139.57–3103.61	31.50–1564.51	110.58–752.50	118.29–1744.19

**Table 3:** Clustering of metabolites sharing similar behavior. Ratios between the pooled metabolites of high HSP27 at baseline and 3-month follow-up (high HSP: 3M/baseline), low HSP27 at baseline and 3-month follow-up (low HSP: 3M/baseline) and high HSP27 and low HSP27 at baseline (baseline: low HSP/high HSP) are clustered on behavior. Ratios between 0.667 and 1.5 are classified as stable.

Behavior	Ratios			Metabolites	Percentage
	High HSP: 3M/Baseline	Baseline: Low HSP/High HSP	Low HSP: 3M/Baseline		
*Normalization*	<0.667	<0.667	Stable	Ribose-5P, Aspartate, beta-Alanine, Hypoxanthine, Oxiglutathione, Pyroglutamic acid	8.43%
	>1.5	>1.5	Stable	Citric Acid	
*Increase*	>1.5	>1.5	>1.5	Creatine P	2.4%
	>1.5	<0.667	>1.5	GDP	
*Decrease*	<0.667	<0.667	<0.667	Inosine	1.2%
*Independent*	<0.667	<0.667	>1.5	Sedoheptulose-7P, Fumarate, CMP, 3-phosphoglyceric acid	31.32%
	<0.667	Stable	Stable	3- and 4-hydroxybenzoic acid	
	>1.5	Stable	Stable	Gluconate-6P	
	>1.5	Stable	<0.667	dAMP	
	Stable	Stable	>1.5	Allantoin, Malate	
	Stable	Stable	<0.667	Coenzyme A, Hippuric acid	
	Stable	<0.667	>1.5	Uracil, UDP-HexNac, 2-phosphoglyceric acid, Adenine, Glycerol-3P	
	Stable	<0.667	Stable	Xanthine, Phosphorylethanolanine, Aminoadicpic acid, Glucose 6P, Glutathione, Ribose	
	Stable	>1.5	Stable	Creatine, Guanosine	
	Stable	>1.5	<0.667	AMP, GMP, Mesaconic acid	
*Stable*	Stable	Stable	Stable	2-aminoisobutyric acid, 2-Dehydrogluconate, 2-Hydroxyglutarate, Alanine, Alpha, Ketoglutarate, Arginine, Asparagine, Citrulline, Creatinine, Cysteine, Cystine, FAICAR, Gluconate, Glucose, Glutamate, Glutamine, Glyceraldehyde-3P, Glycerate, Glycerol-2P, Hexose-P, Hydroxyphenyllactic acid, Isoleucine, Kynurenic acid, Kynurenine, Lactate, Leucine, Lysine, Malonic acid, Methionine, Ornithine, Orotic acid, Pantothenic acid, Phenylalanine, Phosphoenolpyruvate, Proline, Pyruvate, Serine, Succinate, Taurine, Threonine, Tryptophan, Tyrosine, UMP, Uric acid, Uridine, Valine	55,4%

## Supplementary Materials

The following are available online at https://www.mdpi.com/2073-4409/9/7/1729/s1, Table S1: Pooled levels of 83 metabolites of the four groups: high HSP: baseline, high HSP: 3-month follow-up (3M), low HSP: baseline and low HSP: 3M, and the derived ratios; high HSP: 3M/baseline, low HSP: 3M/baseline, baseline: low HSP/high HSP, Table S2: Human pathways of metabolites which show an association between HSP and metabolic energy levels. In addition, the global metabolism pathway which the human pathway corresponds with is denoted (x).

## Appendix A

### Internal Standards for Metabolomics

A 75 µL mixture of the following internal standards in water was used: adenosine-15N5-monophosphate (100 µM), adenosine-15N5-triphosphate (1 mM), D4-alanine (100 µM), D7-arginine (100 µM), D3-aspartic acid (100 µM), D3-carnitine (100 µM), D4-citric acid (100 µM), 13C1-citrulline (100 µM), 13C6-fructose-1,6-diphosphate (100 µM), guanosine-15N5-monophosphate (100 µM), guanosine-15N5-triphosphate (1 mM), 13C6-glucose (1 mM), 13C6-glucose-6-phosphate (100 µM), D3-glutamic acid (100 µM), D5-glutamine (100 µM), 13C6-isoleucine (100 µM), D3-leucine (100 µM), D4-lysine (100 µM), D3-methionine (100 µM), D6-ornithine (100 µM), D5-phenylalanine (100 µM), D7-proline (100 µM), 13C3-pyruvate (100 µM), D3-serine (100 µM), D5-tryptophan (100 µM), D4-tyrosine (100 µM), D8-valine (100 µM).
