# Supplementary material for: Daily Supplementation of L-Glutamine in Atrial Fibrillation Patients: The Effect on Heat Shock Proteins and Metabolites
Source: Cells. 2020 Jul 20;9(7):1729. doi: 10.3390/cells9071729 (PMC7408381; doi:10.3390/cells9071729)
Supplement: Supplementary file 1 [file cells-09-01729-s001.pdf]

Table S1. Pooled levels of 83 metabolites of the four groups; high HSP: baseline, high HSP: 3-month follow-up (3M), low HSP: baseline and low HSP: 3M, and the derived ratios; high HSP: 3M/baseline, low HSP: 3M/baseline, baseline: low HSP/high HSP.

Metabolites	Absolute Values				Ratios		
	High HSP: Baseline	High HSP: 3M	Low HSP: Baseline	Low HSP: 3M	High HSP: 3M/Baseline	Low HSP: 3M/Baseline	Baseline: Low HSP/high HSP
2-aminoisobutyric acid	287781,75	277788,47	285416,84	214518,22	0,97	0,75	0,99
2-Dehydrogluconate	221990,38	231779,98	263663,53	280698,09	1,04	1,06	1,19
2-Hydroxyglutarate	179094,73	184905,70	213122,48	230604,84	1,03	1,08	1,19
2-phosphoglyceric acid	9926,70	8988,38	5199,31	7997,68	0,91	1,54	0,52
3-_or_4-hydroxybenzoic acid	10833,38	7093,30	14676,67	10051,74	0,65	0,68	1,35
3-phosphoglyceric acid	6537,20	1955,93	1596,29	2724,92	0,30	1,71	0,24
Adenine	5423,51	6909,21	2816,32	4277,13	1,27	1,52	0,52
Alanine	716595,75	707790,81	691113,69	696588,19	0,99	1,01	0,96
Allantoin	418144,81	577707,25	325782,84	687823,25	1,38	2,11	0,78
Alpha-Ketoglutarate	940000,81	1047807,62	1086722,12	1196047,88	1,11	1,10	1,16
Aminoadipic acid	82965,83	59265,31	53330,67	52626,80	0,71	0,99	0,64
AMP	6237,84	4863,07	25807,34	10240,82	0,78	0,40	4,14
Arginine	1202491,38	1264341,62	1447160,88	1451369,12	1,05	1,00	1,20
Asparagine	1169169,62	1128355,62	1191380,50	1270973,12	0,97	1,07	1,02
Aspartate	1950403,00	1124820,12	902401,25	863328,75	0,58	0,96	0,46
beta-Alanine	489070,22	300834,41	241404,47	255252,84	0,62	1,06	0,49
Citric acid	553016,31	1132180,12	1209130,12	1394095,38	2,05	1,15	2,19
Citrulline	784433,75	796071,19	771386,75	802437,19	1,01	1,04	0,98
CMP	2983,49	472,36	1466,52	3696,88	0,16	2,52	0,49
Coenzyme_A	1521,25	2052,42	1927,21	919,93	1,35	0,48	1,27
Creatine	245893,25	224660,03	390407,16	299876,72	0,91	0,77	1,59
Creatine-P	2239,47	5574,60	6194,40	10863,28	2,49	1,75	2,77
Creatinine	387146,94	407434,75	415656,44	478292,44	1,05	1,15	1,07
Cysteine	381218,09	479506,28	498019,75	346933,97	1,26	0,70	1,31
Cystine	1505512,50	1754215,38	1973982,12	1428263,25	1,17	0,72	1,31
dAMP	1276,62	2460,60	1875,82	925,48	1,93	0,49	1,47
FAICAR	7713,72	9556,80	8597,92	9202,78	1,24	1,07	1,11
Fumarate	154782,72	99075,59	84258,62	141831,33	0,64	1,68	0,54
GDP	892,85	1712,25	125,81	261,81	1,92	2,08	0,14
Gluconate	1508107,75	1249584,62	1022769,06	1180698,50	0,83	1,15	0,68
Gluconate-6P	9953,55	15665,16	12618,45	11933,20	1,57	0,95	1,27
Glucose	15051773,00	15415444,00	17053558,00	14473830,00	1,02	0,85	1,13
Glucose-6P	48728,48	38391,93	27251,93	39509,27	0,79	1,45	0,56
Glutamate	3777537,75	3276630,00	3258287,50	2892569,75	0,87	0,89	0,86
Glutamine	7359593,50	7269876,00	7231684,50	7353338,50	0,99	1,02	0,98

Glutathione	273734,25	197898,98	169461,56	136910,77	0,72	0,81	0,62
Glyceraldehyde-3P	17259,19	17093,24	11644,60	14950,53	0,99	1,28	0,67
Glycerate	2420490,50	1996610,88	1919937,12	1930657,25	0,82	1,01	0,79
Glycerol-2P	23346,77	19750,82	18253,38	24790,04	0,85	1,36	0,78
Glycerol-3P	109429,25	80145,04	55516,98	87604,36	0,73	1,58	0,51
GMP	6084,78	6083,10	32880,94	17010,21	1,00	0,52	5,40
Guanosine	27225,68	32666,48	46811,73	35511,89	1,20	0,76	1,72
Hexose-P	55519,26	62733,46	57668,94	60660,54	1,13	1,05	1,04
Hippuric acid	5122954,00	5001819,50	4068981,75	2557098,75	0,98	0,63	0,79
Hydroxyphenyllactic acid	391409,44	364428,97	316635,09	403702,75	0,93	1,27	0,81
Hypoxanthine	4133240,75	1126731,00	692743,75	721457,81	0,27	1,04	0,17
Inosine	3740350,25	359541,16	1020823,44	678759,94	0,10	0,66	0,27
Isoleucine	951234,81	862355,00	725410,81	758966,81	0,91	1,05	0,76
Kynurenic acid	16246,47	15923,84	13601,25	19662,87	0,98	1,45	0,84
Kynurenine	14726,31	11901,50	12381,64	16343,15	0,81	1,32	0,84
Lactate	1753591,50	1876363,75	1455605,00	1610763,62	1,07	1,11	0,83
Leucine	499256,72	437421,78	359404,44	384554,25	0,88	1,07	0,72
Lysine	1479301,25	1461074,00	1580014,50	1528886,38	0,99	0,97	1,07
Malate	109803,13	134011,34	137286,62	225086,86	1,22	1,64	1,25
Malonic acid	584030,38	667470,81	785184,00	749892,31	1,14	0,96	1,34
Mesaconic acid	204886,19	235236,62	414397,53	262510,47	1,15	0,63	2,02
Methionine	1032750,94	824053,00	1030812,00	943850,38	0,80	0,92	1,00
Ornithine	1145510,88	1045397,81	961652,12	1025651,00	0,91	1,07	0,84
Orotic acid	31423,60	25836,63	28049,31	28791,13	0,82	1,03	0,89
Oxiglutathione	6445,94	4046,31	2806,63	3108,22	0,63	1,11	0,44
Pantothenic acid	28282,16	22077,56	21334,92	25665,18	0,78	1,20	0,75
Phenylalanine	432958,88	348660,59	356347,62	386391,44	0,81	1,08	0,82
Phosphoenolpyruvate	72120,92	88755,93	81087,99	87339,29	1,23	1,08	1,12
Phosphorylethanolamine	28125,24	27514,03	18592,93	26249,35	0,98	1,41	0,66
Proline	694712,12	590366,69	660961,19	703327,31	0,85	1,06	0,95
Pyroglutamic acid	3732751,25	2007733,75	2020519,00	1925066,12	0,54	0,95	0,54
Pyruvate	3090132,75	3607122,00	2798369,00	2582617,00	1,17	0,92	0,91
Ribose	78844,84	69713,19	41277,18	42491,13	0,88	1,03	0,52
Ribose-5P	67586,32	13006,05	13230,18	16366,28	0,19	1,24	0,20
Sedoheptulose-7P	9813,83	2893,79	2653,62	5372,14	0,29	2,02	0,27
Serine	2069198,12	1662040,88	1715682,88	1721282,50	0,80	1,00	0,83
Succinate	547055,50	496645,81	498179,00	611279,25	0,91	1,23	0,91
Taurine	7920577,00	6883827,00	7242785,00	7073309,50	0,87	0,98	0,91
Threonine	3028374,75	2603921,50	3203242,75	2857039,75	0,86	0,89	1,06
Tryptophan	239878,62	209133,36	230502,98	231175,75	0,87	1,00	0,96
Tyrosine	2083736,50	1488541,62	1578549,38	1490897,12	0,71	0,94	0,76
UDP-HexNac	4607,83	3300,96	804,66	1873,41	0,72	2,33	0,17
UMP	81202,38	89027,72	96067,88	101030,18	1,10	1,05	1,18

Uracil	15774,99	16091,43	8874,64	18312,55	1,02	2,06	0,56
Uric acid	16063145,00	16035150,00	17161386,00	15726882,00	1,00	0,92	1,07
Uridine	797953,38	945954,81	790121,38	788372,06	1,19	1,00	0,99
Valine	2501336,00	2417852,25	2203344,75	2174082,25	0,97	0,99	0,88
Xanthine	2970335,25	2135401,50	1363402,38	1407568,75	0,72	1,03	0,46

Table S2. Human pathways of metabolites which show an association between HSP and metabolic energy levels. In addition, the global metabolism pathway which the human pathway corresponds with is denoted (x).

Behavior	Metabolites	Human Pathways	Global Metabolism Pathways			
			Carbo-Hydrates	Nucleotides	Amino Acids	Vitamins & Cofactors
Normalization	Ribose 5P	Pentose phosphate pathway	x			
		Purine metabolism		x		
		Carbon metabolism	x			
		Biosynthesis of amino acids			x	
	Aspartate	Alanine, aspartate and glutamate metabolism			x	
		Arginine biosynthesis			x	
		Biosynthesis of amino acids			x	
		beta-Alanine metabolism			x	
		Pantothenate and CoA biosynthesis				x
		Carbon metabolism	x			
		2-Oxocarboxylic acid metabolism		x		
		Histidine metabolism			x	
	beta-Alanine	beta-Alanine metabolism			x	
		Histidine metabolism			x	
		Pantothenate and CoA biosynthesis				x
		Pyrimidine metabolism		x		
		Propanoate metabolism	x			
	Hypoxanthine	Purine metabolism		x		
	Oxiglutathione	Glutathione metabolism				
	Pyroglutamic acid	Glutathione metabolism				
	Citric acid	Citrate cycle (TCA cycle)	x			
		2-Oxocarboxylic acid metabolism	x			
		Glyoxylate and dicarboxylate metabolism	x			
		Biosynthesis of amino acids			x	
		Alanine, aspartate and glutamate metabolism			x	
		Carbon metabolism	x			
Increase	Creatine P	Arginine and proline metabolism			x	
	GDP	Purine metabolism		x		

Decrease	Inosine	Purine metabolism	x	
